# Effects of Exergames on Brain and Cognition in Older Adults: A Review Based on a New Categorization of Combined Training Intervention

**DOI:** 10.3389/fnagi.2022.859715

**Published:** 2022-03-30

**Authors:** Marta Maria Torre, Jean-Jacques Temprado

**Affiliations:** Institut des Sciences du Mouvement (ISM), UMR 7287, CNRS, Aix Marseille Université, Marseille, France

**Keywords:** aging, exergames, combined training, physical activity, brain, cognition

## Abstract

The literature on exergames has reported inconsistent benefits on brain and cognitive functions. Moreover, it is still unknown whether they yield to equal or superior benefits as compared to other forms of physical exercise. However, until now, a review of exergaming literature was lacking, that would reverse the “product first” approach to replacing it with a “training first” approach that is, an analysis of the different studies based on a detailed description of the type of combined training interventions that was supported by the utilized exergames. In the present review, thanks to a structured framework build around seven interacting constructs (stimuli, settings, targets, markers, outcomes, moderators, and mechanisms), which collectively afford a global picture of the determining factors of exergames training, we aimed to determine whether and under which conditions exergames could be more effective than conventional training. Twenty three studies were finally selected for review and analyzed. We concluded that, in spite of their potential to improve brain and cognition, beneficial factors contributing to exergaming efficacy as well as its underlying mechanisms need to be investigated more systematically thanks to common experimental designs based on gold standards. We proposed some directions in this respect.

## Introduction

For the growing number of older adults, increased longevity is accompanied by inherent declines in the functioning of different subsystems (i.e., neural, perceptive, cognitive, sensorimotor, cardiovascular, neuro-muscular…), which impact mobility, balance control, motor coordination and, finally, autonomy and quality of life. Fortunately, physical, motor, and cognitive training may attenuate or delay (at least partially) age-related alterations of functional capacities. The question remains, however, of which type(s) of training intervention(s) is/are most effective to improve brain health and cognitive performance in healthy older adults (Diamond and Ling, 2016, 2020; Pesce and Voelcker-Rehage, 2020; Torre and Temprado, 2022).

This issue has been widely addressed with regards to conventional training interventions that is, training programs based on exercises supervised by a coach, without the help of new technologies (e.g., Law et al., 2014; Lauenroth et al., 2016; Zhu et al., 2016; Joubert and Chainay, 2018; Torre and Temprado, 2022). Given the impact of separated physical, motor, and cognitive training on brain health and cognition, it has been suggested that the association of cognitive, motor, and/or physical exercises into combined training interventions might lead to complementary (additive) benefits and, thus, might be a more effective solution (e.g., Bamidis et al., 2014; Desjardins-Crepeau et al., 2016; Diamond and Ling, 2020; Pesce and Voelcker-Rehage, 2020; Dhir et al., 2021; Gavelin et al., 2021). However, due to the heterogeneity of existing studies and the lack of a theoretical framework to put an order in the available literature, inconsistent findings were reported in most reviews (e.g., Lauenroth et al., 2016; Guo et al., 2020 versus Zhu et al., 2016; Gheysen et al., 2018). Recently, we showed that these inconsistencies can be reduced by distinguishing physical-cognitive (PCT), motor-cognitive (MCT), and multi-domain training (MDT) (Torre and Temprado, 2022; see Table 1 below). In doing that, we showed that, regardless of the kind of delivered training intervention, in the (rare) studies in which combined training was well-designed and well-conducted, conventional combined training interventions were more effective than separated training to improve brain and cognition in healthy older adults (see Dhir et al., 2021 for a converging conclusion).

**TABLE 1:** Quality assessment of the selected studies.

PCT						
Anderson-Hanley et al., 2012	Some concerns	High	High	Some concerns	Some concerns	High
Barcelos et al., 2015	Some concerns	High	High	Some concerns	Some concerns	High
MCT						
Park and Yim, 2016	Low	Low	Low	Low	Low	Low
Schoene et al., 2013	Some concerns	Some concerns	High	Low	Some concerns	Moderate
Schoene et al., 2015	Some concerns	High	Low	Low	Low	Moderate
Gschwind et al., 2015	Some concerns	Some concerns	Low	Low	Low	Low
Schättin et al., 2016	Low	Some concerns	Some concerns	Some concerns	High	Moderate
Adcock et al., 2020	Some concerns	High	Some concerns	Some concerns	Low	Moderate
Carrasco et al., 2019	High	High	Some concerns	Some concerns	Some concerns	High
Eggenberger et al., 2015	Some concerns	High	Some concerns	Some concerns	Some concerns	Moderate
Eggenberger et al., 2016	Some concerns	Some concerns	Some concerns	Some concerns	Low	Moderate
Huang, 2020	Some concerns	High	Some concerns	Some concerns	Some concerns	High
Li et al., 2020	Some concerns	High	High	Low	Low	High
MDT						
Kayama et al., 2014	Some concerns	Some concerns	Some concerns	Low	Low	Moderate
Maillot et al., 2012	Some concerns	Some concerns	Low	Some concerns	Some concerns	Moderate
Chuang et al., 2015	High	High	Some concerns	Some concerns	Some concerns	High
Ordnung et al., 2017	Low	Low	Low	Low	Low	Low
Guimarães et al., 2018	Low	Low	Low	Low	Low	Low
Htut et al., 2018	Some concerns	Low	Low	Low	Some concerns	Low
Bacha et al., 2018	Low	Some concerns	Low	Some concerns	Low	Low
Peng et al., 2020	High	High	High	Low	High	High
Moreira et al., 2021	High	Low	Low	Some concerns	Low	Moderate
Gouveia et al., 2021	Low	Low	Some concerns	Some concerns	Some concerns	Moderate
	Bias arising from the randomization process	Bias arising from the randomization process	Bias due to missing	Bias in the measurement of outcomes	Bias in the selection of reported results	Overall bias

*PCT, physical-cognitive training; MCT, motor-cognitive training; MDT, multidomain training.*

This conclusion was consistent with the general hypothesis that physical, motor, and cognitive exercises may have distinct and complementary impacts on brain and cognition in healthy older adults (Voelcker-Rehage et al., 2010, 2011; Chapman et al., 2016, 2017; Raichlen and Alexander, 2017). Specifically, it can be assumed that in well-designed combined training programs: (i) the effects of aerobic training will be magnified when supported by complex motor skills (e.g., Pesce, 2012; Diamond and Ling, 2016, 2019), and (ii) adding highly demanding cognitive stimulations to physical (endurance and muscular resistance) and/or motor exercises will potentiate their effects on brain and cognition (Gheysen et al., 2018). In summary, all the combined training solutions that would enrich physical exercises and motor skills training by increasing their cognitive demands are hypothesized to be more effective than separated training interventions (Torre et al., 2021).

Exergames – i.e., “interactive video-games games that require participants to be physically active to play” (Anderson-Hanley et al., 2017; Stanmore et al., 2017) – are considered a promising solution in this respect (e.g., Costa et al., 2019; Ketelhut et al., 2021). Indeed, ideally, they might capitalize on the widely demonstrated effects of video-games training on brain plasticity and cognitive performance (e.g., Nouchi et al., 2012; Anguera et al., 2013; for overviews, Bediou et al., 2018; Dale et al., 2020), together with the more important role of cognitive demands on the observed benefits of combined training on brain and cognition, relative to physical and motor components (Chapman et al., 2016, 2017; Gheysen et al., 2018).

However, this optimistic view is challenged by inconsistent findings that have been reported in the different reviews and meta-analyses dedicated to exergaming in healthy older adults. Indeed, some of them suggested that exergaming had equal or superior benefits to conventional training (e.g., Howes et al., 2017; Stanmore et al., 2017; Stojan and Voelcker-Rehage, 2019), while others came to the opposite conclusion (e.g., Ogawa et al., 2016; Gallou-Guyot et al., 2020; Sala et al., 2021; Temprado, 2021).

This was the case since existing reviews: (i) primarily focused on the effects of exergames on physical outcomes, balance control, and fall risks (e.g., Larsen et al., 2013; Laufer et al., 2014; Ogawa et al., 2016; Neri et al., 2017), rather than on brain and cognition (for noticeable exceptions, Bleakley et al., 2013; Zhang and Kaufman, 2016; Stanmore et al., 2017; Stojan and Voelcker-Rehage, 2019), (ii) mixed both healthy and diseased older adults (e.g., Bleakley et al., 2013; Stanmore et al., 2017), or (iii) did not distinguish between sedentary video games and exergames (Zhang and Kaufman, 2016; Vázquez et al., 2018; Mansor et al., 2019). In addition, the authors of the few reviews that addressed the effects of exergames on cognitive performance and brain functioning mentioned the great heterogeneity of the selected studies concerning frequency, duration, the intensity of training sessions, or cognitive demands of exergames (e.g., Stanmore et al., 2017; Stojan and Voelcker-Rehage, 2019). We contend, however, that a more important reason is that most studies were conducted according to a “product first” approach, which aimed to test the effectiveness of specific exergaming products (either off-the-shelf or lab-customized) (e.g., Maillot et al., 2012; Chao et al., 2014; Laufer et al., 2014; Amjad et al., 2019; Carrasco et al., 2019; Li et al., 2020), instead of considering the kind of combined training that was delivered, regardless of the utilized exergame. However, exergaming is characterized by its versatile combination possibilities of aerobic, strength, and cognitive exercises into various multicomponent training modes (Stojan and Voelcker-Rehage, 2019). Specifically, according to our previous categorization (Torre and Temprado, 2022), different types of combined training programs – i.e., physical-cognitive, motor-cognitive, or multi-domain training – can be delivered through the use of similar products (e.g., Xbox Kinect, Nintendo Wii, Dance Dance Revolution…), depending on intervention settings (e.g., exercise duration, variability of exercises…), body movements or the games that are utilized. However, by neglecting this issue, the majority of studies missed the main moderator of training effectiveness that is, the type of combined training programs effectively proposed to participants. In addition, most reviews on exergames were few, if any, interested in the cognitive contents of digital environments, though their features may make them more or less effective to stimulate the brain and cognition (Bediou et al., 2018; Dale et al., 2020; Temprado, 2021). Thus, until now, a review of exergaming literature was lacking, that would reverse the “product first” approach to replacing it with a “training first” approach that is, an analysis of the different studies based on a detailed description of the type of combined training interventions that was supported by the utilized exergames.

The present review is a step in this direction. Indeed, it re-analyzed the available literature on exergames thanks to a new categorization of combined training (i.e., physical-cognitive, motor-cognitive, and multi-domain) and a structured framework, which were previously applied to review the literature on conventional combined training interventions (Torre and Temprado, 2022). Thanks to this framework, we aimed to determine whether and under which conditions, the benefits of exergaming were equal or superior to those of conventional training. A secondary objective was to critically appraise exergame studies, identify the still unanswered questions, and delineate new directions to guide future research and the design of new exergames.

## A Framework for Analyzing Combined Training Intervention Delivered via Exergames

**FIGURE 1:**
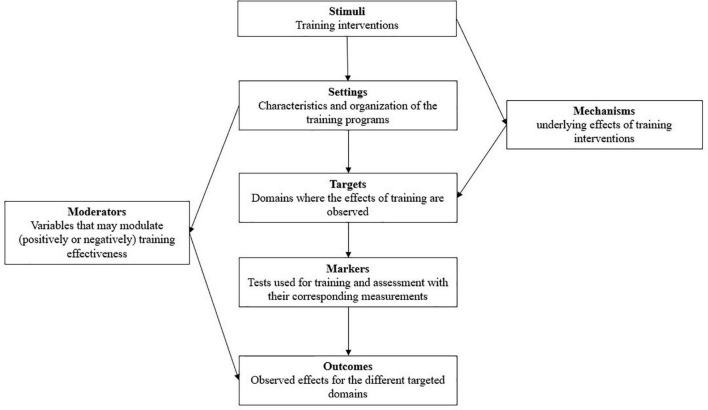
A structured framework of combined training with exergames. Detailed explanations are provided in the text.

To do the present review, based on the existing knowledge on physical, motor, and cognitive training, we developed a structured framework including seven constructs to afford a detailed picture of the determining factors of training effectiveness. Specifically, our framework distinguishes: (i) the stimuli, (ii) the settings, (iii) the targets of training, (iv) the markers, (v) the outcomes, (vi) the moderators, and (vii) the underlying mechanisms (Figure 1). In this respect, it is more detailed than the classic PICO procedure (Population, Intervention, Comparison, Outcomes), which is currently used in other reviews (e.g., Soares et al., 2021).

### Stimuli

This construct refers to the type of training delivered to the participants, independent of the utilized exergame solution. Assuming that exergames allow combining cognitive stimulations with physical and/or motor exercises, we categorized the reviewed studies according to three types of associations (Torre and Temprado, 2022): (i) physical-cognitive training (PCT), which corresponds to the combination of cognitive stimulations with endurance (aerobic) and/or muscular resistance training; (ii) motor-cognitive training (MCT), which refers to the combination of complex motor skills training and additional cognitive stimulations, with low cardio-vascular (i.e., endurance) effort, and (iii) multi-domain training (MDT), which consists of associating (at least moderate) cardio-vascular (endurance) effort, complex motor skills training, and additional cognitive stimulation.

### Settings

For the different studies, we determined whether the exergaming group was compared to either a control group (active/inactive) or different conventional, separated, or combined training groups. In addition, we noted whether the studies included transfer tests and/or retention tests after the intervention period. Then, we carefully analyzed how the training programs were organized and conducted, according to general “FIT-VP” principles of training effectiveness, namely: the frequency of sessions (F), the intensity of physical exercises (I), time (T) (duration of sessions and training program), variety of exercises (V) and progressivity of training load (P). By crossing the frequency of sessions, their duration, and the duration of intervention, the volume of training proposed in each study was calculated. Also, importantly, we tried to determine whether the intervention was supervised, the type of instructions communicated to participants, whether and how feedbacks were given to participants, whether training loads were progressively increased or, eventually, individualized. If possible, we identified the games used in the different studies and their cognitive contents.

### Targets

Primary targets referred to the investigated cognitive processes and, in some cases, brain activity, structures, or neurobiological mechanisms impacted by training. Secondary targets were physical and motor capacities related impacted by training (balance control, gait, mobility, endurance, muscular force…).

### Markers

Markers refer to the tests used, pre-and post-intervention, to assess the targeted functions and capacities, with their corresponding measured variables (e.g., response time, errors…).

### Outcomes

Outcomes correspond to the observed effects of training for the different targeted domains. Our analysis focused primarily on the impacts on brain and cognition, but training-related changes in physical fitness and/or motor fitness were also considered to assess the effectiveness of physical and/or motor training.

### Mechanisms

When mentioned in the reviewed studies, we identified the theoretical frameworks, models, or specific physiological, neurobiological, and psychological mechanisms that were proposed to explain the observed results.

### Moderators

This construct refers to the variables that were explicitly identified in the different studies as modulating (positively or negatively) training effectiveness. They could be related to the settings of training interventions (duration of the training program, frequency of sessions, duration of sessions, supervision by a coach…), while others could be related to the characteristics of participants (age, gender, education, lifestyle, performance baseline, fitness level, adherence to the program…), methodology (intent to treat, the distinction between responders and not responders…) or of the utilized exergame.

## Methods

### Search Strategy

The present study was guided by the Preferred Reporting Items for Systematic Review and Meta-Analysis guidelines (PRISMA). It consisted of re-analyzing Randomized Controlled Trials (RCT) and Controlled Trials (CT) selected from the reviews and meta-analyses dedicated to exergames and published, in English, from 2010 to July 2021. In addition, recently published RCT/CT (from July 2021 to November 2021), not mentioned in the selected reviews, were also identified and screened.

Exergames were defined as solutions that “combine physical and cognitive exercise in an interactive digital, augmented, or virtual game-like environment” (Stojan and Voelcker-Rehage, 2019). Accordingly, activities performed in sitting conditions and controlled with handheld devices (i.e., sedentary video games) were not included. Also, fitness games (e.g., Fitlight Training™ or similar devices), which do not include video games interfaces, were not considered as exergames (for example, see Torre et al., 2021).

The first step of the analysis consisted of identifying the reviews and meta-analysis of interest, through systematic searches conducted in Medline, Science Direct, PsychINFO, and Google Scholar. Search terms were (“review” OR “meta-analysis”) AND (“exergam*” OR “virtual reality” OR “active video game”) AND (“aging” OR “aging” OR “older adults” OR “healthy older adults”) AND “English.” A second step consisted of carefully analyzing the different studies cited as references in the included reviews and meta-analysis, to select a subset of studies among those cited. Only RCT and CT were considered. Unpublished articles, thesis, dissertations, or book chapters were discarded. Finally, a third step consisted of identifying the studies published between July 2021 and November 2021 through similar systematic searches (“combined training” OR “cognitive-motor” OR “dual-task training” OR “multicomponent training” OR “multidomain training”) AND (“older adults” OR “healthy older adults” AND “English”). No study met the inclusion criteria.

### Selection Process and Data Extraction

Reviews and meta-analyses identified after the systematic searches were screened by title, abstract and content relevance by two independent researchers (MT and J-JT). They were considered relevant if they met the following criteria: (i) concerning the effects of exergames on brain and cognition, and (ii) including only healthy older adults (>60 years old) or a separated analysis of healthy older adults among other populations. Each researcher should select “included,” “excluded,” or “doubt” and describe the reason for doubt or exclusion. Subsequently, the researchers compared the answers and discussed each disagreement to define the articles to be read in full text. In case of doubt, the full text was used for a final decision.

**FIGURE 2:**
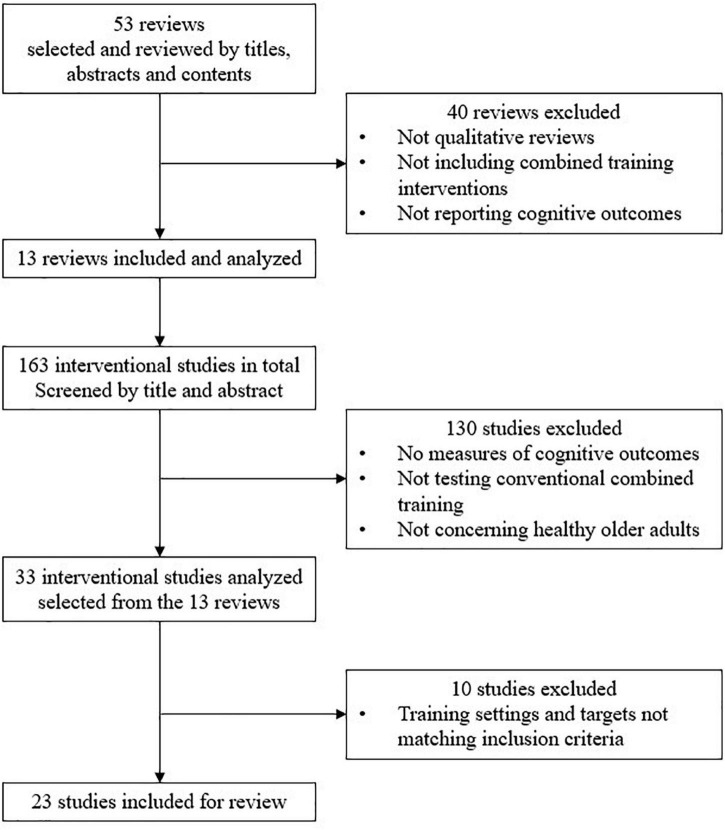
Flow chart of the selection process.

Thirteen reviews and meta-analyses (out of 53 initially screened) were selected based on these criteria (see Table 2). Then, we selected the most relevant studies among those cited in the 13 reviews and meta-analyses. One hundred and sixty-three not redundant references were screened. To be finally included in our review, the RCT and CT had: (i) to concern exergames (i.e., not sedentary videogames), (ii) to consist in a training program lasting at least 4 weeks, (iii) to include one session of 30 min or more per week, (iv) to report primary or secondary outcome measures to capture at least one of the following domains: (i) cognitive functions, (ii) global cognition [e.g., Mini-Mental State Examination (MMSE), Montreal Cognitive Assessment (MoCA)], or (iii) brain functional or structural data. The lack of measurement of physical (VO2, muscular strength.) and motor outcomes (balance control, walking speed, motor coordination…) was not considered a reason for exclusion, though it might preclude the assessment of the effects of training on physical and motor domains, as possible moderators of benefits on cognition. On the other hand, we did not include the studies in which only physical (muscular force, Vo2max…) or motor outcomes (balance control, gait…), cognition in everyday life, motivation, well-being, stress, depression, or anxiety were measured. Studies reporting analyses of brain activity were considered relevant only if they concomitantly reported cognitive outcomes. Studies that included dual-task cost measures during gait or postural tasks, but no separated assessment of cognitive processes (EF, attention, memory, …), were considered marginally relevant and then, not included. Finally, 23 studies were selected for review (see Figure 2 and Table 2). Quality assessment of the different studies has been carried out using the Cochrane RoB assessment tool (Table 1).

**TABLE 2:** Summary table of selected reviews and studies.

Selected reviews	PCT	MCT	MDT
Zhang and Kaufman, 2016	**Sequential**		
Bleakley et al., 2013		Park and Yim, 2016	Kayama et al., 2014
Ogawa et al., 2016	**Simultaneous**		
Howes et al., 2017	Anderson-Hanley et al., 2012	Schoene et al., 2013	Maillot et al., 2012
Stanmore et al., 2017	Barcelos et al., 2015	Schoene et al., 2015	Chuang et al., 2015
Vázquez et al., 2018		Gschwind et al., 2015	Ordnung et al., 2017
Mansor et al., 2019		Schättin et al., 2016	Guimarães et al., 2018
Stojan and Voelcker-Rehage, 2019		Adcock et al., 2020	Htut et al., 2018
Gallou-Guyot et al., 2020		Carrasco et al., 2019	Bacha et al., 2018
Wollesen et al., 2020		Eggenberger et al., 2015	Peng et al., 2020
Gavelin et al., 2021		Eggenberger et al., 2016	Moreira et al., 2021
Sakaki et al., 2021		Huang, 2020	Gouveia et al., 2021
Soares et al., 2021		Li et al., 2020	

## Results

### Overview of Combined Training Interventions

We categorized the selected studies as a function of the type of association between physical, motor, and cognitive stimulations, which was proposed via the utilized exergame (see Table 3).

**TABLE 3:** Summary of the criteria used to categorize the type of combined training in different studies on exergames.

	**Physical-cognitive training:** Endurance effort and/or muscular resistance exercises + cognitive-demanding virtual environment	**Motor-cognitive training:** Complex motor skills + cognitive-demanding virtual environment		**Multi-domain training:** Endurance effort and/or muscular resistance exercises + complex motor skills + cognitive-demanding virtual environment	

Sequential		Conventional training program (Park and Yim, 2016)	Rowing on Kayak in a virtual reality environment. Water training simulated. Moving kayaks were directly filmed in a river and a lake. The subjects exercised by paddling according to watch the actions performed by the 3-D images on the screen (Park and Yim, 2016).	Conventional motor training program (Kayama et al., 2014). Conventional functional fitness exercises: marching in place, step touches, stepping on pads, and squats.	Microsoft Kinect motion capture device – A new game consisting of Dual-Task Tai-Chi tasks (Kayama et al., 2014). Xbox Kinect V2 sensor. Five customized exergames (i.e., “Grape Stomping,” “Rabelos,” “Exermusic,” “Toboggan,” “Ride,” and “Exerpong”) which cover aerobic training, muscular resistance exercises and balance control exercises (Gouveia et al., 2021).
Simultaneous	Stationary bike + virtual reality tour or high cognitive-demanding game (collecting coins through spatial navigation in the virtual landscape) (Anderson-Hanley et al., 2012; Barcelos et al., 2015)	Virtual Fruit Ninja game. Equipped with a sword, the player must slice fruits while carefully avoiding the bombs (Huang, 2020). Interactive step training incorporating a modified version of DDR training and a choice reaction time task (Schoene et al., 2015) Microsoft Xbox Kinect. Balance exergames (i.e., walking, stepping, weight shifting) and strength exercises based on the Weight-bearing Exercise for Better Balance (WEBB) program (Gschwind et al., 2015). Virtual Dancing Game combining an attention demanding cognitive task with a motor coordination task (Eggenberger et al., 2015, 2016; Schättin et al., 2016) “Active@Home.” A technology-based game to train strength, balance and cognition through Tai Chi-inspired exercises, dancing, and step-based cognitive games (Adcock et al., 2020). Nintendo Wii. Sports games (Tennis, Baseball, Bowling, and Boxing) (Carrasco et al., 2019). Multi-tasking-based Virtual Reality motion video game – “Whac-A-Mole” game. The players to move all limbs to feed animals as they emerge from their holes (Li et al., 2020)		Nintendo Wii. Wii Sports, Wii Fit and Mario & Sonic on Olympic Games (Maillot et al., 2012). Konami Dance Dance Revolution, consisting of stepping coordinated movements and aerobic training (Chuang et al., 2015). Microsoft Xbox 360 Kinect. Multi-domain video exergame training combining endurance, coordination, strength as well as demands on cognitive processing (Ordnung et al., 2017). Microsoft Xbox Kinect. Sport Ultimate Games (Bowling, Boxing, Skiing, Soccer, Table Tennis, Tennis, and Track and Field), together with “mini games” of shorter duration (<1 min) (e.g., Pin Rush, Target Kick, Super Saver, Body Ball, and Paddle Panic) (Guimarães et al., 2018; Htut et al., 2018). Microsoft Xbox Kinect. Kinect Adventures games stimulating fast movements and center of gravity control, through multidirectional steps, squats, jumps, coordinated movements of the upper and lower limbs, and trunk movements in three planes. The games also include fast decision making, environmental monitoring, inhibition of responses, and divided attention (Bacha et al., 2018). Interactive lab-customized exergame (EMAT). A ladder-type, three-by-three grid-type, and circle type mat exergame with simultaneous cognitive–physical training (Peng et al., 2020). Microsoft Xbox Kinect. “Your Shape Fitness” games: strength exercises (squats and lunges), balance and cardiorespiratory exercises (boxing and lateral and antero-posterior displacements) (Moreira et al., 2021). Xbox Kinect V2 sensor. Five customized exergames (i.e., “Grape Stomping,” “Rabelos,” “Exermusic,” “Toboggan,” “Ride,” and “Exerpong”) which cover aerobic training, muscular resistance exercises and balance control exercises (Gouveia et al., 2021).	

*The corresponding studies and the exergames utilized are listed.*

Physical-cognitive training studies were those in which cognitive stimulations, delivered through 3D or virtual reality environments, were associated with low to moderate levels of cardio-vascular effort in task situations that neither involved complex full-body movements, nor upper-limbs complex coordinated movements. This was typically the case in studies that associated cognitive challenges and pedaling on a stationary bike. MCT studies included the studies in which training situations required performing complex full-body movements, stepping tasks, and/or upper-limbs coordinated movements, in association with cognitive stimulations presented on the digital environment. Thus, in these studies, muscular force and motor fitness were often assessed in addition to cognitive functions. On the other hand, aerobic effort was scarcely of interest and, in general, its level was neither controlled during training nor assessed pre-and post-intervention. MDT studies proposed training situations, which incurred low to moderate levels of cardio-vascular effort, currently reached through a combination of full-body movements, stepping tasks, and/or upper-limbs coordinated movements, while simultaneously performing cognitive challenges thank a 3D, video game interface. In these studies, cardio-vascular effort was often controlled during the training sessions. Moreover, progress in endurance capacities and motor fitness were frequently measured post-intervention, in addition to cognitive performance.

Notably, however, in a few studies, these criteria were only partially met, due to uncertainties concerning the level of aerobic effort and/or muscular force incurred by the training situations. Indeed, in most cases, information was lacking to evaluate this aspect either during training sessions or concerning the effects of training on cardio-vascular and/or muscular capacities. Similarly, progresses in exergame-based outcomes (e.g., scores) were never reported in the different studies, thereby precluding any assessment of the efficiency of training in this domain, independent of the different outcomes observed on the targeted functions. In these few cases, the two reviewers decided to attribute the study in one of the three categories based on the information provide about the exercises and utilized exergame, with the help of a third reviewer (JL), specialized in exercise physiology, to estimate the intensity of aerobic effort.

### Overview of Utilized Exergames

The exergames used in the various studies were balanced between classic commercial products offered by players in the video game market (i.e., Nintendo Wii, Microsoft Xbox Kinect, Expresso Cyber-Cycle, Konami Dance Dance Revolution) and new solutions, also inspired from commercial exergames, but conceived by research groups and developed by specialized start-ups (15 and 7 studies, respectively, see Table 3). Among the commercial products, the Xbox Kinect was the most frequently used exergame, mainly to implement MDT.

### Physical-Cognitive Training

#### Stimuli

Only two studies implemented pure physical–cognitive training (Anderson-Hanley et al., 2012; Barcelos et al., 2015). Both used a recumbent, stationary bike (i.e., the cyber-cycle Expresso™), which allowed to control and record the duration and intensity of aerobic exercise while interacting with 3D gamified environments displayed on a screen. The interaction was operated thanks to movements of small amplitudes consisting of lifting/pushing the handles (i.e., up/down), located on either side of the saddle.

#### Settings

In the two studies, physical exercise consisted of an endurance effort. The Heart Rate Reserve (HRR) method was used to control the intensity of exercise. Participants were asked to maintain their heart rate at 60% of their HRR. Sessions were not supervised but instructions were given to participants by a clinician during a familiarization period (1 month), to help them learning to use the cyber-cycle. Ride behavior was recorded thanks to feedback information provided by the bike device (mileage, Kcal…).

Anderson-Hanley et al. (2012) compared a cyber-cycle group and a control group. In the cyber-cycle condition, pedaling allowed the participants to move on a 3D virtual bike tour displayed on a screen, to compete with their own “ghost” rider. After 2 months, they were instructed to outpace on-screen riders, which presumably corresponded to an increase in the level of aerobic effort. In the control group, participants pedaled on a traditional stationary bike, viewing heart rate and mileage as feedback information. Using a similar exergame, Barcelos et al. (2015) assigned participants either to a condition of physical exercise plus low cognitive demand (similar to the cyber-cycle condition used by Anderson-Hanley et al., 2012) or to a condition of physical exercise plus high cognitive demand resulting from spatial navigation in a 3D gamified environment to collect different-colored coins and corresponding-colored dragons. Presumably, playing the game required higher cognitive engagement than the simple bike tour (i.e., more planning, multi-tasking, and strategizing), though no detailed analysis of the cognitive contents of both situations was provided. In both studies, training programs lasted 3 months. Sessions lasted 20 up to 45 min, with a frequency of two times per week (Barcelos et al., 2015) up to five (Anderson-Hanley et al., 2012; Barcelos et al., 2015). Participants were tested pre-and post-intervention, but not in long-term follow-up.

#### Targets

In both studies, the targets of training interventions were identified through pre- and post-intervention assessments. In both studies, executive functions (attention, inhibition, and short-term memory) were the primary targets for which a composite score was obtained by converting raw scores on each test to z-scores using the grand mean and SD across both groups for each time point, then averaging the three measures. In addition, Anderson-Hanley et al. (2012) included secondary cognitive targets (verbal fluency, verbal memory), while Barcelos et al. (2015) only measured general cognitive status to characterize the sample of participants, at baseline. Measures of physiological (weight, fat mass…) and motor (visuospatial manual skills) variables also allowed to characterize the sample of participants. Since no changes were expected on these tests, no post-intervention assessment was carried out. Notably, no measures of changes in physical fitness (e.g., 6 min walking test, sit-to-stand test…) were carried out. The daily level of physical activity (in Kcal) was measured during the training period by Anderson-Hanley et al. (2012). Ride behaviors (frequency, intensity, and duration) were recorded by participants on a paper log. Barcelos et al. (2015) also recorded participants’ self-ratings of their everyday functioning in regards to memory and concentration. Finally, in Anderson-Hanley et al. (2012) targeted neurotrophic factors through the analysis of BDNF concentration in blood plasma, pre-and post-intervention (i.e., not after each session).

#### Markers

Similar tests (i.e., the Color Trails 1 and 2, the Stroop C, and the Digit Span tests) were used in the two studies to assess the executive functions.

#### Outcomes

In both studies, post-intervention differences were observed between the two groups for the composite score of executive functions. On the other hand, no difference was observed for the secondary outcomes (attention, verbal fluency, verbal memory, or visuospatial skills) (Anderson-Hanley et al., 2012). Notably, in Anderson-Hanley et al.’s (2012) study, the difference between the two groups likely resulted from lack of training effects in the conventional cycling condition (even, a decline of cognitive functions), while a medium positive effect was observed in the cyber-cycle condition. In Barcelos et al.’s (2015) study, the training effect was larger in the high demanding condition than in the low demanding condition, only for the Stroop C task.

An increase in blood concentration of BDNF was observed in the participants of the cyber-cycle condition when compared to the traditional exercise group (Anderson-Hanley et al., 2012). Also, cyber-cyclists experienced a 23% reduction in risk of clinical progression to MCI compared with traditional exercisers (i.e., nine controls versus three cyber-cyclists converted to MCI).

#### Mechanisms

Anderson-Hanley et al. (2012) proposed several mechanistic explanations for their results. On the one hand, they hypothesized that larger cognitive benefits observed in the cyber-cycling condition might reflect the added mental exercise required by the virtual reality experience. It might result either from navigating in a 3D landscape, anticipating turns, and competing with others, thereby stimulating attentional focus, divided attention, and decision making, and/or from dual-task situations provided by these environments. Another explanation, proposed in both studies, referred to neurobiological underpinnings of the combination of physical and cognitive exercises in the cyber-cycling condition, which would stimulate the mechanisms of brain plasticity (angiogenesis, neurogenesis), and other changes that foster neurovascular integrity. According to this hypothesis, cognitive stimulation would add to the blood release of BDNF, when compared to conventional physical intervention.

#### Moderators

The moderators of the observed outcomes were not included in the discussion of the two studies. Concerning the clinical/functional status of the participants, both focused on healthy older adults. The age of participants was different in the two studies, namely: equal or superior to 55 years old for Anderson-Hanley et al. (2012; mean age = 78), and older in Barcelos et al.’s (2015) study (mean age = 82 years) with a majority of women, which could be a possible moderator. Anderson-Hanley et al. (2012) admitted that the age and education of the participants were unequal, thereby precluding generalizability of the results. Notably, a significant group difference was found between the exergaming and the control group for education, which might also take part in the observed results.

In both studies, after a period of familiarization, the training programs were not supervised, which could be a possible moderator of global effectiveness. For instance, given that the cyber-cycle condition was experienced as “fun” by participants, it could be that it was more motivating than the control condition, especially in the absence of a supervisor to maintain enjoyment.

The programs lasted 3 months during which the participants were instructed to freely increase the frequency and duration of sessions, to reach five times per week and 45 min/session (Anderson-Hanley et al., 2012). However, no indication was provided about the criteria used to increase the difficulty of exercises. Also, how many participants reached five sessions/per week at the end of the program was unclear. Barcelos et al. (2015) mentioned that 58% of the participants were partially adherent, while 42% fully adhered to the minimum dose of their assigned exergaming condition during the 3-month. In Anderson-Hanley et al.’s (2012) study, 21% of the enrolled participants did not complete the study. How many sessions finally did these participants remain unknown. Also, whether intention-to-treat was used or whether these participants were excluded from the analyses was not mentioned. Finally, no measures of physical fitness were carried out to assess age-related changes in aerobic capacities, which is a weakness of the study.

In both studies, since physical and cognitive stimulations were delivered simultaneously, training doses were equivalent in the two domains. No differences in exercise frequency, intensity, or duration were found between the different groups. Finally, the progress of participants in the exergame-based outcomes was not reported in either of the two studies.

#### Discussion

The results reported by Anderson-Hanley et al. (2012) suggested that, all conditions being equal, PCT delivered via a cyber-cycle exergame may lead to larger enhanced cognitive and neurobiological benefits than conventional physical training on a stationary bike or, at least, may allow limiting age-related declines in cognitive functions. This conclusion could be valid only in healthy older adults. Indeed, using quite similar exergames and conditions, Karssemeijer et al. (2019) did not observe any superiority of exergaming over classic aerobic training on the stationary bike in people with dementia. Moreover, the results observed by Barcelos et al. (2015) suggested that the benefits of training with the cyber-cycle strongly depended on the cognitive challenge provided by the gamified environments (i.e., low and higher cognitively demanding conditions). Notably, however, the benefits of cyber-cycling might be observed only for a subset of EF (e.g., flexibility and inhibition). This result suggested that the gamified environment selectively loaded these functions.

Benefits of exergaming were observed even though the endurance effort was of low intensity in the different conditions. These results suggest that, for PCT, the added value of the cyber-cycle (and more generally, PCT delivered via exergaming) could not only consist of “enriching” physical training with cognitive challenges (Gheysen et al., 2018) but, also, decreasing the level of endurance effort needed to improve cognitive functioning via physical training, thanks to a highly demanding cognitive stimulation (see Pesce, 2012; Diamond and Ling, 2016, 2020; for converging conclusion). However, it is noticeable that in Anderson-Hanley et al.’s (2012) study, no benefits on executive functions were observed for the conventional training group, which is not consistent with previous research on conventional aerobic training and suggests that the intensity of physical effort was too low to trigger any benefits, even if it led to release of BDNF in the blood circulation. Instead, the observed results suggested that cognitive training was dominant over physical training, which has previously been observed in the literature on conventional combining training (Torre and Temprado, 2022). According to this interpretation, exergaming with the cyber-cycle would consist of cognitive training, rather than PCT. However, since no information was provided, in both studies, about changes in physical capacities after training, and since no separated cognitive training groups were included in the two studies, this hypothesis remains plausible but speculative.

Though Anderson-Hanley et al.’s (2012) study studies did lend credence to the superiority of physical-cognitive training delivered via an exergame (i.e., a cyber-cycle) over conventional aerobic training, limitations precluded identifying the conditions of effectiveness of the cyber-cycle (or an equivalent product), which remain to be explored in future studies. First of all, whether PCT delivered via exergaming could be more effective to improve brain and cognition than separated (intense enough) physical, motor (i.e., complex motor skills), or cognitive training remains unknown. Thus, a more complete experimental design, including different training groups could be recommended. Moreover, in the two studies, the cognitive contents of the gamified environments associated with the cyber-cycle were neither precisely identified nor quantified, so that it remains unclear whether the used virtual environments stimulated dual-task mechanisms and/or spatial navigation processes or any other processes. These issues could be addressed in future studies.

### Motor-Cognitive Training

#### Stimuli

Eleven studies typically consisted of motor–cognitive training (MCT) that is, interventions in which aerobic effort was very low and, accordingly, physical outcomes (e.g., cardio-vascular and endurance capacities) were not mainly targeted. Instead, MCT principally consisted of a short bout of exercises delivered thanks to different exergames, which had in common to require balance control, weight-bearing and stepping in different directions, or walking in place. This was the case for: (i) the multi-component in-home training exergame – Active@Home, which included Tai Chi-inspired exercises, dancing, and step-based cognitive games (Adcock et al., 2020); (ii) the SMT sensor platform connected to gamified environments (e.g., StepMania, Stepper, Trail-stepping, and Tetris), which allowed participants to produce stepping sequences with various levels of difficulty in step patterns and frequency (Schoene et al., 2013, 2015; Eggenberger et al., 2015, 2016; Gschwind et al., 2015), (iii) lab-customized balance games (i.e., KIN, Gschwind et al., 2015; Whac-A-Mole; Li et al., 2020; 3D Kayak program, Park and Yim, 2016), (iv) the commercial Microsoft Xbox 360 Kinect and Nintendo Wii Fit exergaming packages (Carrasco et al., 2019; Huang, 2020), and (v) the Impact Dance Platform, which required the participants to perform specific whole body movements, driven by a video game presented on a frontal screen (Schättin et al., 2016). Three studies used similar exergames (i.e., the SMT; Schoene et al., 2013, 2015; Gschwind et al., 2015). Specifically, Schoene et al. (2013, 2015) investigated the effectiveness of the “SMT” exergaming platform relative to an inactive control group, while Gschwind et al. (2015) compared the “SMT” and the “KIN” exergames.

Few details were provided about the cognitive contents of the gamified environments associated with the different exergames. Sometimes, the nature and general principles of the utilized games were globally described (Eggenberger et al., 2015, 2016; Park and Yim, 2016; Carrasco et al., 2019; Huang, 2020; Li et al., 2020), in particular when they consisted of lab-customized video games (Schoene et al., 2015; Schättin et al., 2016). On the other hand, the details of stimulated functions were not systematically indicated (Schoene et al., 2013, 2015; Gschwind et al., 2015; Schättin et al., 2016; Adcock et al., 2020). This was consistent with the “product first” approach, according to which the authors were confident with the global effectiveness of technologies, *per se*.

#### Settings

Over the 11 studies on MCT, one associated sequentially conventional motor exercises (stepping, walking, one-leg standing…) and exergaming (i.e., VR Kayaking) (Park and Yim, 2016), within each session. In the remaining studies, exergaming was compared either to an inactive control group (Schoene et al., 2013, 2015; Carrasco et al., 2019; Adcock et al., 2020; Li et al., 2020) or to active control groups, which were trained conventionally (e.g., balance training, stepping.; Park and Yim, 2016; Schättin et al., 2016) and via another exergame (Gschwind et al., 2015). Also, Huang (2020) compared two exergaming groups, which were trained with a similar game (“Fruit Ninja”), one displayed with a classic 3D environment and the other displayed with an immersive, virtual reality-based environment. Only one study compared exergaming with two other groups following conventional physical aerobic training (walking on a treadmill) and cognitive (i.e., memory) training, respectively (Eggenberger et al., 2015).

Intervention lasted either 4 weeks (Huang, 2020; Li et al., 2020), 6 weeks (Eggenberger et al., 2015; Park and Yim, 2016; Carrasco et al., 2019), 8 weeks (Schoene et al., 2013; Eggenberger et al., 2016; Schättin et al., 2016), 16 weeks (Schoene et al., 2015; Adcock et al., 2020) or even 26 weeks (i.e., 6 months, Eggenberger et al., 2015). Session frequency was quite similar in the different studies (2/3 per week), while their durations were between 20/30 min (Schoene et al., 2013, 2015; Gschwind et al., 2015; Eggenberger et al., 2016; Schättin et al., 2016; Adcock et al., 2020; Huang, 2020; Li et al., 2020) up to 1 h (Eggenberger et al., 2015; Park and Yim, 2016; Carrasco et al., 2019). In all studies, participants were tested pre- and post-intervention. Only one carried out a 1-year follow-up (Eggenberger et al., 2015). Thus, the total duration of effective practice was between 4 h (Huang, 2020) and 26 h (Eggenberger et al., 2015).

#### Targets

Executive functions, in particular, cognitive flexibility and inhibition, were the most frequently targeted cognitive processes (Schoene et al., 2013, 2015; Eggenberger et al., 2015, 2016; Gschwind et al., 2015; Schättin et al., 2016; Carrasco et al., 2019; Adcock et al., 2020; Huang, 2020; Li et al., 2020), together with attentional processes (Eggenberger et al., 2015; Schoene et al., 2015; Adcock et al., 2020; Li et al., 2020). Memory and information processing speed were less frequently targeted (Eggenberger et al., 2015).

On the motor side, the targeted outcomes were: (i) balance control (Gschwind et al., 2015; Park and Yim, 2016; Adcock et al., 2020; Li et al., 2020), (ii) motor coordination and stepping (Schoene et al., 2013, 2015; Adcock et al., 2020), or (iii) gait and mobility (Schoene et al., 2013; Schättin et al., 2016; Adcock et al., 2020). Muscle strength of lower limbs was assessed in two studies through the Sit-to-Stand test (Schoene et al., 2013; Gschwind et al., 2015; Adcock et al., 2020). In one study, the Physiological Profile Assessment (PPA) allowed testing proprioception, reaction time, postural sway, the strength of lower limb, mobility, and endurance (Schoene et al., 2013; Adcock et al., 2020).

Two studies tested training-related changes in brain functions, thanks to fNIRS and EEG (Eggenberger et al., 2016; Schättin et al., 2016), and one in brain volume, thanks to MRI (Adcock et al., 2020). Psychological outcomes (fear of falling, well-being, anxiety, depression, quality of life) were measured in a few studies (Schoene et al., 2013, 2015; Huang, 2020). However, results are not reported since this domain is outside the scope of the present study.

#### Markers

A large number of different tests were used in the ten selected studies. Moreover, several studies used similar tests to assess the different targeted cognitive capacities (see Tables 4, 5).

**TABLE 4:** List of markers used in the different studies to target cognitive functions (MCT).

Cognitive processes	Markers	Studies (MCT)
General cognitive status	MoCa	Eggenberger et al., 2016; Park and Yim, 2016
Executive control	Victoria Stroop Test Trail Making Test Part A-B Executive Control Task, Adaptive n-Back Test Reaction time for information processing speed	Gschwind et al., 2015; Schoene et al., 2015; Eggenberger et al., 2016; Adcock et al., 2020; Huang, 2020 Schoene et al., 2013, 2015; Eggenberger et al., 2015, 2016; Carrasco et al., 2019; Adcock et al., 2020; Huang, 2020 Eggenberger et al., 2015, 2016; Li et al., 2020 Adcock et al., 2020
Attention	Attentional Network Test Age Concentration Test A and B	Gschwind et al., 2015; Adcock et al., 2020 Eggenberger et al., 2015; Schättin et al., 2016; Li et al., 2020
Memory	Paired Associated Learning Task, Wechsler Memory Scale, Digit Forward and Backward Rey’s Complex Figure Test Digit Span	Eggenberger et al., 2015 Carrasco et al., 2019 Schoene et al., 2015; Huang, 2020
Reasoning	Raven’s Standard Progressive Matrices	Gschwind et al., 2015; Li et al., 2020

**TABLE 5:** List of the markers used in the different studies to target physical and motor functions (MCT).

Physical and motor functions	Markers	Studies (MCT)
Upper and lower limb muscle strength	Handgrip Strength, Arm Curl Test Five Sit to Stand Test	Park and Yim, 2016 Schoene et al., 2013; Gschwind et al., 2015; Adcock et al., 2020
Dexterity Agility – Mobility	Time Up and Go Timed Up and GO in Dual Task Choice Stepping Reaction Time Victoria Stroop Stepping Task (Cognitive – Motor Resources)	Schoene et al., 2013; Gschwind et al., 2015; Adcock et al., 2020 Schoene et al., 2013; Schättin et al., 2016; Adcock et al., 2020 Schoene et al., 2013 Schoene et al., 2015
Static and dynamic balance	Single-Leg Stand Test, Standing and Sitting Balance Alternate Step Test	Park and Yim, 2016; Li et al., 2020 Schoene et al., 2013
General physical-motor status	Short Physical Performance Battery Physiological Profile Assessment	Eggenberger et al., 2016; Adcock et al., 2020 Schoene et al., 2013

Also, there was large heterogeneity in the tests used to assess motor capacities. Balance control, gait, and mobility were the most frequently assessed motor capacities. proprioception, muscular strength, and endurance capacities were scarcely assessed. Some studies tested cognitive-motor resources underlying behavioral outcomes (see Tables 4, 5).

#### Outcomes

##### Motor-Cognitive Training Versus Control Group

In the five studies that compared an exergaming group and a passive control group (Schoene et al., 2013, 2015; Carrasco et al., 2019; Adcock et al., 2020; Li et al., 2020), results showed a significant benefit of exergaming for information processing speed (Adcock et al., 2020), divided attention (Carrasco et al., 2019; Adcock et al., 2020), working memory (Li et al., 2020), and executive functions (Carrasco et al., 2019). Performance in dual-task was also positively impacted (Adcock et al., 2020).

For physical outcomes, no significant improvements got evident for any physical functions (i.e., force or aerobic endurance) in any study (Schoene et al., 2015; Carrasco et al., 2019; Adcock et al., 2020; Li et al., 2020). To motor outcomes, positive effects were observed for the 30 s chair rises test (Adcock et al., 2020), the one-leg balance test (eyes open) (Li et al., 2020), the choice stepping reaction time test (Schoene et al., 2013, 2015) and physiological profile assessment and the dual-task TUG and verbal fluency task (Schoene et al., 2013). No increase in brain volume was found after exergaming (Adcock et al., 2020). Instead, brain volume decreased in both the exergaming and control groups.

##### Motor-Cognitive Training Versus Conventional Physical/Motor Training

Two studies compared an exergaming training group and a conventional motor training group (Eggenberger et al., 2016; Schättin et al., 2016), while Eggenberger et al. (2015) compared exergaming and physical (aerobic) training (i.e., walking on a treadmill). This was done, for the exergaming groups, thanks to interactive video-game-based allowing the participants to perform specific whole-body movements driven by different games presented on a frontal screen. Game exercises are required to combine attention-demanding cognitive tasks and complex coordinated movements (Eggenberger et al., 2015, 2016; Schättin et al., 2016).

Eggenberger et al. (2016) and Schättin et al. (2016) observed that both the conventional training and exergaming programs resulted in enhanced performance of several cognitive functions (i.e., cognitive flexibility, inhibition, working memory, or divided attention). However, no superior benefits of exergaming were observed. Notably, in Schättin et al.’s (2016) study, performance improvement after exergaming was observed in all the measured functions, while it was observed only for cognitive flexibility in the conventional training group. In both the conventional training and exergaming groups, improvements in cognitive performance correlated with a reduction in PFC activity during walking at preferred and fast velocities, which resembled young-adults brain functions (Schättin et al., 2016; Eggenberger et al., 2016). No difference in the reduction of brain activity was observed between the two groups by Eggenberger et al. (2016), while Schättin et al. (2016) reported larger effects in the exergaming group. Also, exergaming was found more effective than balance training to improve walking in dual-task situations (Schättin et al., 2016). Taken together, these results were interpreted by the authors as a reduction of the need for executive function and attention involved in challenging treadmill walking after both conventional training and exergaming.

Eggenberger et al. (2015) found superior benefits of exergaming over conventional aerobic training (walking) on shifting attention and working memory. Notably, no difference was found between the two training programs for the different markers of physical fitness (i.e., the short physical battery test and 6 min walking).

Finally, Park and Yim (2016), using a sequential protocol associating conventional balance training and VR Kayak exergame, showed larger improvements than conventional balance training only on muscle strength, and balance control in both sitting and standing positions. A benefit was also observed on general cognition, but no additional measurements to target specific processes were carried out.

##### Motor-Cognitive Training Versus Cognitive Training

Eggenberger et al. (2015) compared the effects of exergaming and cognitive (memory) training on different cognitive functions (shifting, working memory, and episodic memory). They found superior benefits of exergaming on working memory.

##### Comparison 2 Exergames Delivering Motor-Cognitive Training

Gschwind et al. (2015) compared two exergames that are, one based on training lower limbs muscular force and balance control, thanks to stepping and weight-bearing exercises (KIN), and another (SMT), which stimulated cognitive functions presumably involved in fall risks (i.e., divided attention, inhibition, processing speed, choice stepping reaction time). As expected, the SMT group improved proprioception, reaction time, sit-to-stand performance, and executive functioning, while the KIN group only improved muscle strength. Huang (2020) compared two exergames based on “Fruit Ninja,” but delivered thanks either to an immersive (IVR) or to a non-immersive (VR) exergame. The results showed larger benefits of IVR on inhibition and task-switching (measured by the Stroop Test and Trail Making Test, respectively), which were mediated by the sentiment of presence created by the IVR exergame.

#### Moderators

The moderators of the effects observed in the different studies were scarcely analyzed, *per se*, by the authors. Sometimes, the limitations of the study were mentioned in the discussion. Moreover, due to the heterogeneity of the protocols of the different studies, it was difficult to identify common features of settings, which could characterize effective motor-cognitive exergaming programs. At least, it seems that associating sequentially in each session, conventional balance control exercises and exergaming (in this order) into a training program (Park and Yim, 2016) was an efficient solution to develop both physical and cognitive capacities. However, this hypothesis is based on the results of only one study.

Concerning the utilized exergames, cognitive contents were scarcely described. According to Huang (2020) study, the sentiment of presence generated (or not) by immersive conditions could be a strong moderator of the effects on cognitive functions.

Baseline cognitive status could also be a potential moderator of the observed outcomes. Indeed, larger benefits were observed in the participants with poorer baseline performance (see Schoene et al., 2015, for supporting evidence). Education, motivation, training enjoyment, gender, daily living cognitive and physical activities, or anthropometric characteristics of participants could also be possible moderators but they were not considered as such in most studies, as soon as no difference was observed between the exergaming group and the other groups. Sample sizes of participants and the choice of outcome measurements were also mentioned as possible moderators (Adcock et al., 2020). Actually, in the selected studies, samples sizes were ranged from 10 participants/group (Li et al., 2020) to 74 (Gschwind et al., 2015), with very different intermediate values in the other studies (i.e., 16, 18, 22, 33, 36, 37, and 45). In a few studies, sample sizes were calculated about the expected statistical power. The attrition rate, which was ranged from 0% (Eggenberger et al., 2015) to 20–30% (e.g., Eggenberger et al., 2016; Adcock et al., 2020) should also be considered as a possible moderator of the results. Analyzing completers and not completers would be of interest, though not done. Indeed, considering only the participants that have completed the program, instead of intention-to-treat, may introduce a bias toward positive benefits. Even, within these participants, initial fitness level might be considered, together with high/low adherers, responders and not responders, which might be distinguished and analyzed separately (see Schoene et al., 2015; Temprado et al., 2019; for a convincing analyses in this respect). Last but not least, the role of motivation, which could be different in the exergaming and the conventional training conditions was never mentioned as a possible moderator.

In most studies, effective dose-response relationships were difficult to assess since the total duration of exercises effectively performed by the participants was not reported (for a noticeable exception, Schoene et al., 2015), in particular when the interventions were not supervised (e.g., Schoene et al., 2013, 2015; Eggenberger et al., 2015, 2016; Gschwind et al., 2015; Schättin et al., 2016; Adcock et al., 2020; Li et al., 2020). However, effective training doses may play a role in the observed outcomes. For instance, the superiority of the SMT exergame over the KIN exergame reported by Gschwind et al. (2015) might result from the longer mean duration of practice of the former relative to the latter (4.5 h and 12.7 h, respectively). Conversely, the lack of effects of exergaming observed by Schoene et al. (2013) and Carrasco et al. (2019) might result from a too-short training program (8 h and 12 h), although Huang (2020) observed benefits of exergaming after only 16 h of training. Adcock et al. (2020) also speculated that training durations were not long enough to trigger an increase in brain volume, as frequently observed for longer (i.e., 6–12 months) training programs, including those delivered thanks to exergames (Ji et al., 2017; Anderson-Hanley et al., 2018). Whether exergaming intervention was supervised or not is important to consider, as a possible moderator but it was never done in the different studies.

Training load and its progressive increase over time is also a widely considered moderator of training effectiveness, in both physical and cognitive domains (Schättin et al., 2016; Adcock et al., 2020). However, objective quantifications of cognitive and physical training loads were rarely provided in the different studies, and the criteria used to progressively increase training load, when done, were not detailed (for a noticeable exception, Adcock et al., 2020). In addition, whether the participants progressed in the exergame-based outcomes was never mentioned in the reviewed studies.

Finally, a last but not least (possible) moderator could be the used exergame itself. For instance, commercial exergames were more or less adequately designed for older adults and were more of less immersive. This possibility was however never explicitly envisaged in the different studies either since the authors were blindly confident in the efficiency of their product (be it a commercial one or a lab-customized exergame), or since they mainly aimed to test the efficiency of the used exergame, without deeply questioning the underlying mechanisms at work during training.

#### Mechanisms

Hypotheses about the mechanisms underlying the synergistic effects of cognitive and motor training stimulations were scarcely provided in the different studies. Adcock et al. (2020) measured changes in brain volume according to the theoretical assumption that the combination of motor and cognitive exercises might stimulate neurobiological mechanisms underlying neuroplasticity, which was in turn considered the mediator of increases in cognitive performance. However, since they didn’t observe any change in brain volume after training, while the performance of executive functions improved, this explanation falls short, presumably since the motor training load was too low. No alternative mechanism was proposed. Eggenberger et al. (2015) also evoked the role of neurobiological mechanisms triggered by the combination of motor and cognitive training as, for instance, increased neurogenesis and synaptogenesis in the cortical structure, promotion of cerebral metabolism, alterations of neurotransmitter and neurotrophic factor levels, availability of cerebral oxygen and glucose, and reduced oxidative stress. Since in their study, the level of aerobic exercise was very low, it can be hypothesized that the observed benefits of exergaming on “shifting attention” and memory resulted from strength (Marinus et al., 2019) and coordination training (Voelcker-Rehage et al., 2010, 2011).

Eggenberger et al. (2016; see also Schättin et al., 2016) showed that changes in brain activity in the PFC resulting from exergaming were related to better performance of executive functions. These findings suggested that combined training delivered thanks to the used exergame was able to reduce the need for prefrontal resources of executive function and attention involved in challenging walking. This hypothesis is consistent with the results observed by Li et al. (2020), which underlined the potential of exergames to improve attentional mechanisms and dual-tasking abilities.

#### Discussion

The results of the studies on MCT with exergames suggested that compared to a passive control group: (i) exergaming may improve performance in different cognitive domains (for a noticeable exception see Schoene et al., 2013), and (ii) the most sensitive processes were executive functions and working memory. However, attention, information processing speed, and dual-task performance during walking may also be positively impacted by exergaming. Interestingly, this was observed independent of the exergame utilized, to the extent that it combined balance control and cognitive training thanks to virtual 3D environments or video games.

On the other hand, the benefits of exergaming on motor and physical fitness were weak or, even, absent. It might be the case since the level of physical (aerobic) activity incurred during exergaming was low, due to the nature of performed motor activities. Nevertheless, these results suggested that, relative to inactivity, the benefits of the MCT via exergames resulted from the cognitive stimulations delivered thanks to the gamified, virtual reality environments, even if the level of physical exertion or the complexity of the motor skills involved in the different exergames were low, as previously observed for PCT. Thus, one can hypothesize that in most studies, exergaming did not correspond to combined training but, mainly, to cognitive training (weakly if any) potentiated by a low level of physical activity. In support of this assumption, Eggenberger et al. (2015) did not found superior benefits of exergaming on cognitive (executive) functions over separated cognitive (memory) training. Thus, it can be concluded that, since the required motor skills were not complex enough and physical (i.e., aerobic) effort was low, exergaming benefits were closer to those of cognitive training than of combined cognitive-motor training, as initially expected from exergames. Whether MCT with exergames would lead to larger benefits than cognitive training, if motor exercises were more demanding, remains to be investigated in future studies.

In this context, the potential superiority of MCT with exergames over conventional physical and motor training remains unclear. Indeed, two studies did not observe superior benefits of exergaming on executive functions (Eggenberger et al., 2016; Schättin et al., 2016), while larger impacts of exergaming were observed in another study on shifting attention and working memory, when compared with separated physical (aerobic) training (Eggenberger et al., 2015). Also, Gschwind et al. (2015) showed that exergaming was more effective than conventional motor training delivered thanks to a video game interface when it allowed loading executive functions. In this respect, the role of immersive environments was highlighted (Huang, 2020; see Temprado, 2021 for a converging conclusion). On the other hand, whether exergaming may be more effective than conventional motor training to improve dual-tasking capacities (during walking) and to reduce brain level activity in the PFC remains uncertain, due to inconsistent results reported in the only two studies that addressed this issue (Schättin et al., 2016; Eggenberger et al., 2016).

Finally, though encouraging in some ways, the available studies on motor-cognitive training delivered with exergames did not allow identifying the optimal settings, which could lead to greater benefits of exergaming, relative to conventional training. First of all, studies are lacking, which used a complete design with several groups (i.e., motor, cognitive, and combined training). Moreover, our results suggested that exergames were either not designed optimally (e.g., the required motor skills were not complex enough) or not used adequately by participants to deliver real motor-cognitive combined training. For instance, whether participants did not perform the motor exercises as required was impossible to determine since movement kinematics was never recorded and analyzed. However, one could hypothesize that during exergaming, due, for instance, to the dual-tasking situations, a priority was given by the participants to cognitive processing of the information delivered by the virtual environment to the detriment of the quality of execution of the motor exercises. In this respect, whether qualitative feedback about movement execution was provided to participants to guide them to efficient execution remains unknown. To overcome this problem, the results reported by Park and Yim (2016) suggested that an efficient solution consists of associating exergaming sequentially with conventional (complex) motor training. Further studies are however necessary to determine whether larger benefits would be systematically observed with such types of settings, relative to training with exergames only.

In any case, it can be concluded from the above analysis that further studies should be carried out to determine the conditions of the superiority of MCT delivered via exergames, relative to conventional cognitive, motor, and physical training.

### Multi-Domain Training

#### Stimuli

Ten studies were categorized as MDT. They all had in common to combine aerobic, motor, and cognitive exercises through exergaming, at least in one group. Most of the used exergames involved complex, upper-limb, lower-limb and whole-body movements. In a few studies, games were categorized as a function of the physical/motor capacities they prominently required (i.e., endurance, strength, motor coordination, motor ability, balance control…) (e.g., Maillot et al., 2012; Ordnung et al., 2017; Bacha et al., 2018; Guimarães et al., 2018; Gouveia et al., 2021; Moreira et al., 2021; for an illustration, Peng et al., 2020). On the other hand, their cognitive contents were scarcely precisely described (for an exception, Bacha et al., 2018).

Different commercial products were used to implement MDT. The Xbox Kinect console (+ Kinect sensors allowing to control an avatar) was the most frequently used (Kayama et al., 2014; Ordnung et al., 2017; Bacha et al., 2018; Guimarães et al., 2018; Htut et al., 2018; Moreira et al., 2021), together with the Nintendo Wii package (Maillot et al., 2012) and Dance Dance Revolution (Chuang et al., 2015). Lab-customized exergames were also used in two studies (Peng et al., 2020; Gouveia et al., 2021). In Peng et al.’s (2020) study, they consisted of a mat allowing participants to perform steps to play a puzzle game, competing against each other to hit each light on the grid to turn it “off” the most quickly, to play a reaction time game with a basketball or performing verbal memory exercises on the circle-type mat for balance and agility training. In Gouveia et al.’s (2021) study, it consisted of a set of five customized exergames (i.e., “Grape stomping,” “Rabelos,” “Exermusic,” “Toboggan,” “Ride,” and “Exerpong”), each covering the main training domain (e.g., aerobic endurance, upper/lower strength, and motor ability).

#### Settings

Two studies used a sequential combination of exergaming and conventional standardized training programs (aerobic, muscular, and balance control/motor ability) (Kayama et al., 2014; Gouveia et al., 2021). In Kayama et al.’s (2014) study, participants of the exergaming group received five additional minutes of training with a dual-task exergame, consisting of solving sudoku, while performing Tai Chi-like full-body movements. In Gouveia et al.’s (2021) study, participants were exposed to an intervention associating conventional multidomain training (one session per week) and exergaming (one session per week), while the participants from the other group practiced two sessions of conventional multidomain training per week. Thus, only eight studies compared a pure exergaming group with one or several other training groups, that is: (i) a passive control group (Maillot et al., 2012; Ordnung et al., 2017), (ii) an endurance training group (Guimarães et al., 2018), or (iii) a conventional physical-motor training group (Bacha et al., 2018; Peng et al., 2020; Moreira et al., 2021). One study compared the exergaming group with three other groups (conventional physical-motor training, brain training, and passive control) (Htut et al., 2018), and another one compared the exergaming group with two other groups (passive control and brisk walking training) (Chuang et al., 2015). So, in summary, four studies allowed to compare an exergaming group with a passive control group, four studies with a conventional combined training group, two studies with a separated physical (aerobic) training group, and one study with a brain/cognitive training group. Thus, surprisingly, no studies compared exergaming with their conventional counterpart that is, training programs combining physical, motor, and cognitive exercises (e.g., Van het Reve and de Bruin, 2014; for a review, see Torre and Temprado, 2022). Only one study investigated brain activity (Chuang et al., 2015).

Training programs lasted either 6 weeks (Ordnung et al., 2017), 7 weeks (Bacha et al., 2018), 8 weeks (Htut et al., 2018), or 12 weeks for all the other studies, with a frequency of 2/3 sessions of 45 min/1 h per week. Thus, training programs included 14 to 36 sessions, with training duration ranging between 18 h and 36 h. The exergaming sessions were either supervised (Maillot et al., 2012; Kayama et al., 2014; Ordnung et al., 2017; Peng et al., 2020; Gouveia et al., 2021) or not (Chuang et al., 2015; Bacha et al., 2018; Guimarães et al., 2018; Htut et al., 2018; Moreira et al., 2021).

#### Targets

Executive functions and global cognition were the most frequently tested cognitive domains. Visuospatial functions, verbal fluency, short-term memory, and long-term memory, reasoning, information processing speed were also targeted in a few studies. The main targeted physical capacities were endurance, flexibility, upper- and lower limb muscle strength. Balance control was the most frequently tested motor capacity. The other motor capacities were psychomotor reactivity and agility, mobility, and gait speed, motor coordination. Cognitive-motor resources were tested in one study thanks to dual-task walking situations. Brain EEG activity was recorded in only one study (Chuang et al., 2015).

#### Markers

Several tests were used to assess the targeted cognitive functions but few studies used similar tests to assess them (see Table 6). Physical and motor tests were also used in the different studies (for exceptions, Guimarães et al., 2018; Gouveia et al., 2021; see Table 7).

**TABLE 6:** List of markers used in the different studies to target cognitive functions (MDT).

Cognitive processes	Markers	Studies (MDT)
General cognitive status	MoCa MMSE Phone Screening	Kayama et al., 2014; Bacha et al., 2018; Htut et al., 2018; Peng et al., 2020 Guimarães et al., 2018; Moreira et al., 2021 Gouveia et al., 2021
Executive control	Stroop Color Word Interference Test, Letter Sets Test, Matrix Reasoning Test, Digit Symbol Substitution Test Trail Making Test Part A-B Delta Trail Making Test Go-No Go reaction time tasks Flanker task Groton Maze Learning Test	Maillot et al., 2012 Maillot et al., 2012; Kayama et al., 2014; Moreira et al., 2021 Kayama et al., 2014 Ordnung et al., 2017 Chuang et al., 2015 Guimarães et al., 2018
Attention	Identification Test for visual attention	Guimarães et al., 2018
Memory	Groton Maze Recall Test, One Card Learning Test	Guimarães et al., 2018
Psychomotor and perceptual processing speed	Simple and choice reaction time tasks Plate Tapping Test, Comparison Test	Ordnung et al., 2017; Guimarães et al., 2018 Maillot et al., 2012
Visuospatial capacities	Spatial Span task, Directional Heading Test, Mental Rotation Test	Maillot et al., 2012
Dual-tasking	Walking while counting, backward in increments of three from a random number between 90 and 100 walking while carrying a tray that was 80% full of water	Peng et al., 2020
Verbal fluency	Verbal Fluency Test (VFT)	Kayama et al., 2014

**TABLE 7:** List of the markers used in the different studies to target physical and motor functions (MDT).

Physical and motor functions	Tests	Studies (MDT)
Cardio-respiratory fitness - endurance capacities	Senior Fitness Test 2 min Step Test, Borg Category Ratio Scale Submaximal Cycle Ergometer Test Six-Minute Step Test (6MST) 3 Min Step Test	Peng et al., 2020 Htut et al., 2018 Chuang et al., 2015 Bacha et al., 2018 Maillot et al., 2012; Ordnung et al., 2017
Upper and lower limb muscle strength	Senior Fitness Test 30 s Chair Stand Test Handgrip Strength Arm Curl Test, Upper Body Muscular Endurance Test	Peng et al., 2020 Ordnung et al., 2017; Htut et al., 2018 Maillot et al., 2012 Ordnung et al., 2017
Flexibility	Senior Fitness Test Chair Sit and Reach Test, Back Scratch Test	Peng et al., 2020 Maillot et al., 2012; Ordnung et al., 2017; Htut et al., 2018
Dexterity Agility – Mobility	Jebson-Taylor Hand Function Test (JTT) Time Up and Go/Time up and go-cog, Foot Tapping Test Functional Gait Assessment (FGA) Gait speed (4 m at comfortable gait speed and 10 m Walk Test at maximum gait speed)	Ordnung et al., 2017 Htut et al., 2018; Peng et al., 2020 Bacha et al., 2018 Maillot et al., 2012; Moreira et al., 2021
Static and dynamic balance	Single-Leg Stand Test Berg Balance Scale (BBS) Mini-Balance Evaluation Systems Test Wii balance board	Peng et al., 2020 Htut et al., 2018 Bacha et al., 2018 Ordnung et al., 2017

#### Outcomes

##### Multi-Domain Training Versus Passive Control Group

Four studies compared an exergaming group and a control group (Maillot et al., 2012; Chuang et al., 2015; Ordnung et al., 2017; Htut et al., 2018). They all reported significant improvement of the tested cognitive functions – executive functions (Maillot et al., 2012), global cognition (Htut et al., 2018), attention (Ordnung et al., 2017) and processing speed (Maillot et al., 2012; Chuang et al., 2015; Ordnung et al., 2017) – compared to the inactive control group: Concerning physical and motor outcomes, significant benefits were reported for aerobic capacity (Maillot et al., 2012), muscle strength (Maillot et al., 2012; Htut et al., 2018), balance control (Maillot et al., 2012; Htut et al., 2018) and motor coordination (Ordnung et al., 2017).

##### Multi-Domain Training Versus Conventional Physical Training

Two studies compared an exergaming and a physical (aerobic) training group (Chuang et al., 2015; Guimarães et al., 2018). They reported positive effects of the two types of interventions on both cognition (executive function, short-term memory, delayed recall, and global cognition) and cardio-vascular capacities (Chuang et al., 2015), but no superiority of exergaming over conventional training. Even, in Guimarães et al.’s (2018) study, exergaming only impacted executive function and delayed recall, while aerobic training improved the performance of executive functions, short-term memory, delayed recall, and global cognition.

##### Multi-Domain Training Versus Conventional Physical-Motor Training

Six studies compared MDT delivered via exergames with conventional physical-motor training, that is, training programs associating aerobic effort, muscular resistance training, and (more or less) complex motor skills (Kayama et al., 2014; Bacha et al., 2018; Htut et al., 2018; Peng et al., 2020; Gouveia et al., 2021; Moreira et al., 2021).

In general, as expected, both exergaming and conventional training enhanced physical fitness and motor capacities that is, muscular force (Htut et al., 2018; Peng et al., 2020), endurance (Bacha et al., 2018; Peng et al., 2020), flexibility, mobility and balance control (Bacha et al., 2018; Htut et al., 2018; Moreira et al., 2021), though no superiority of exergaming was found on these capacities (excepted Peng et al., 2020). Moreover, results showed that the two kinds of training positively impacted either global cognition (i.e., MoCa, MMSE) or sub-domains (memory, inhibition, information processing speed) (Kayama et al., 2014; Bacha et al., 2018; Gouveia et al., 2021; Moreira et al., 2021). Nevertheless, in four studies, no superiority of exergaming on cognitive domains was observed (Bacha et al., 2018; Peng et al., 2020; Gouveia et al., 2021; Moreira et al., 2021). Conversely, Kayama et al. (2014) reported greater benefits of training with an exergame based on motor-cognitive dual-task training on TMT scores, relative to conventional physical-motor training. This was also observed by Htut et al. (2018), who found that exergaming enhanced cognition (MoCA) to a greater extent than conventional training, while the inverse was observed for muscular force, mobility, and balance control. Notably, Peng et al. (2020) observed superiority of exergaming on dual-task during walking, but not on global cognition (MoCA).

##### Multi-Domain Training Versus Cognitive Training

Htut et al. (2018) compared the benefits of exergaming and those observed after a cognitive/brain training, consisting of Chinese checkers, Jenga, and Match Pair games played collectively. They observed a larger improvement of global cognition and dual-task walking (TUG-cog) after cognitive training than after exergaming. A superiority of exergaming over cognitive/brain training was only observed for muscular force (i.e., Sit-to-Stand test).

#### Moderators

The role played by potential moderators was scarcely discussed in the different studies. Concerning age, all studies included healthy older adults over 60 years. Gender was never evoked as a possible moderator, including in Chuang et al.’s (2015) study in which only female participants were included. More generally, even when the numbers of different groups were unbalanced, there were no separate analyzes for men/women since no specific hypotheses were proposed for gender-dependent effects of exergames.

Training programs lasted between 6 weeks (Ordnung et al., 2017; Bacha et al., 2018; Htut et al., 2018), 8 weeks or 12 weeks (Kayama et al., 2014; Chuang et al., 2015; Guimarães et al., 2018; Peng et al., 2020; Gouveia et al., 2021; Moreira et al., 2021) with one (Kayama et al., 2014; Peng et al., 2020), two (Maillot et al., 2012; Ordnung et al., 2017; Bacha et al., 2018; Gouveia et al., 2021) or three sessions per week (Chuang et al., 2015; Guimarães et al., 2018; Htut et al., 2018; Moreira et al., 2021). Session duration lasted 30/45 min (multiple times a week) (30 min: Chuang et al., 2015; Htut et al., 2018; 45 min: Guimarães et al., 2018; Gouveia et al., 2021; Moreira et al., 2021), up to 60 min (Maillot et al., 2012; Ordnung et al., 2017; Bacha et al., 2018), 75/80 min (Kayama et al., 2014), or even 2 h (Peng et al., 2020). Thus, the number of effective hours of practice was between 4 h (Htut et al., 2018) and 24 h (Guimarães et al., 2018). Only one study (Kayama et al., 2014) used a sequential presentation of only 5 min of practice of exergaming followed by conventional training (which was enough to observe superior benefits of the group practicing exergaming). Thus, in general, MDT with exergames referred to the combination of simultaneous exercises.

Less clear in few studies was whether exergaming was supervised or not (e.g., Bacha et al., 2018; Guimarães et al., 2018), though it might play a critical role (a strong bias), especially in cases where conventional training would be supervised and exergaming, would not. Whether such a situation existed among the different studies was difficult to determine.

Attendance reported only in the different studies was high and it doesn’t seem to have played a critical role in the observed outcomes (i.e., >80%).

Finally, an important potential moderator could be the nature of the utilized games. Indeed, beyond their cognitive contents, some games can incite higher levels of enjoyment and motivation during play than others (see Chuang et al., 2015 for a converging conclusion).

#### Mechanisms

No study on MDT delivered via exergames directly investigated underlying neurobiological mechanisms (e.g., blood release of neurotrophic factors) and only one was interested in brain activity (Chuang et al., 2015). Thus, in the rare studies in which underlying mechanisms were evoked, only speculative hypotheses could be proposed (e.g., Chuang et al., 2015 for an illustrative example). Anyway, in case where there is no difference between exergaming and conventional physical-motor training, one could conclude that, at least, the mechanisms related to physical/motor exercises were at work in exergaming (i.e., neural plasticity) and played a dominant role over those related to cognitive stimulation (e.g., constant monitoring of the screen, planning and quick strategic decision, and adapting to changes in the challenges proposed by the game…) (Guimarães et al., 2018). Conversely, when exergaming showed a superiority, relative conventional training, one could conclude that mechanisms related to cognitive stimulation constituted the added value of exergaming, even if their exact nature remains unknown.

#### Discussion

The results of the different studies on MDT with exergames showed enhanced performance in different cognitive domains, and especially, executive functions, memory, attention, information processing speed, and, even, global cognition. Notably, exergaming also had significant benefits on physical and motor outcomes (endurance, muscular force, balance control, mobility…). Though encouraging, these results just confirmed that “doing something is always better than inactivity.”

Concerning the superiority of exergaming over conventional training, results were balanced. Indeed, in four studies, no superiority of exergaming over conventional physical training was established, for cognitive functions (Chuang et al., 2015; Bacha et al., 2018; Guimarães et al., 2018; Moreira et al., 2021), while greater benefits of exergaming were observed in three studies (Kayama et al., 2014; Htut et al., 2018; Peng et al., 2020). It might be larger benefits of exergaming depending on whether the used exergames were mainly based on dual-task training or not. This hypothesis remains however speculative since details of the cognitive contents of the exergames used in all the different studies were not provided.

The lack of superiority of exergaming over cognitive training was more surprising (Htut et al., 2018). Indeed, it suggested that MDT via exergames failed to capitalize on the additivity of the separate effects of each type of exercise, relative to cognitive training.

## General Discussion

Since they combine cognitive training with video games and physical/motor exercises, exergames are frequently considered a more promising solution than conventional physical training to improve brain and cognition, and more generally, to prevent the effects of aging on the different functional subsystems (e.g., Stanmore et al., 2017; Stojan and Voelcker-Rehage, 2019; Soares et al., 2021). However, in the few reviews that addressed the benefits of exergames on cognitive outcomes, inconsistent results were reported (e.g., Stanmore et al., 2017 versus Sala et al., 2021) or, at least, low to very low quality of evidence was reported (e.g., Howes et al., 2017). Thus, the main motivation of the present work was to carry out, for the first time to our knowledge, a review of the studies on exergames: (i) in healthy older adults, (ii) based on a “training first” approach, and (iii) focusing on their effects on brain health and cognition. To achieve this objective, we analyzed selected studies, thanks to a categorization of combined training intervention and a structured framework, both previously applied to analysis of the literature on conventional combined training (Torre and Temprado, 2022). The main conclusions of this analysis are summarized in the following.

### Correspondence Between Combined Training Modes and the Utilized Exergames

Among the three modes of combined training, two were dominant in the selected studies that is, MCT and MDT (11 and 10 studies each) while only two studies concerned PCT (see Table 3). The exergames used in the different studies corresponded to the proposed training modes. Specifically, for PCT it was a stationary cyber-cycle, while for MCT, the exergames used primarily targeted balance capacities, in particular through short sequences of stepping and weight shifting activities, which were also supposed to develop lower limb strength. More rarely, the exergames used for MCT consisted of upper and lower limb coordination tasks and minimal physical effort. On the other hand, for MDT, the used exergames all required whole-body coordinated movement, which were practiced over longer durations than for MCT. Independent of the training mode, commercial products (i.e., Microsoft Xbox Kinect package, Konami Dance Dance Revolution, and Nintendo Wii Fit package) were most frequently used (15 studies), while lab-customized exergames, principally dedicated to stepping and balance control were used in the other studies. These results confirmed that it mades sense to analyze the different training modes, rather than mixing the exergames as in most reviews, which led to them being seen as delivering comparable exercises (for a converging point of view, see Stojan and Voelcker-Rehage, 2019, p. 15 and 19).

### Effectiveness of Exergame Training to Enhance Brain Functioning and Cognitive Performance

Though it has been defined elsewhere as a golden standard to assess the superiority of combined training interventions over separated ones (Torre and Temprado, 2022), none of the selected studies compared exergaming to physical, motor cognitive and combined training (+ a control group). Moreover, two studies used a sequential training procedure, in which the exergame was proposed for only minutes in addition to traditional exercise, within the same session.

• *Exergaming versus passive control group*

Nine studies out of 23 (five on MCT and four on MDT) investigated the effects of exergaming relative to a passive control group (Maillot et al., 2012; Schoene et al., 2013, 2015; Chuang et al., 2015; Ordnung et al., 2017; Htut et al., 2018; Carrasco et al., 2019; Adcock et al., 2020; Li et al., 2020). They all reported significant improvement of the tested cognitive functions – global cognition, attention, working memory, executive functions and, most frequently, processing speed, except for Schoene et al. (2013), who reported no effect on cognitive flexibility, probably due to the low training charge and sessions frequency of their protocol. This was globally confirmed in the other studies, which compared exergaming and conventional training (see below), and showed within group enhancement of cognitive outcomes. Notably, however, listing the cognitive processes that were impacted in the different studies gives a misleading picture, since positive effects differed greatly across studies and showed inconsistencies. Thus, it was impossible to determine whether a given exergame training mode had specific or larger effects on some cognitive functions than the others. In this respect, our findings are in line with a previous review on exergaming (Stojan and Voelcker-Rehage, 2019), even though we included seven additional studies.

• *Exergaming versus conventional physical and motor training*

Six studies (one on PCT, three on MCT, and two on MDT) compared the effects of exergaming and those of conventional physical (four studies) or motor (two studies) training (Anderson-Hanley et al., 2012; Chuang et al., 2015; Eggenberger et al., 2015, 2016; Schättin et al., 2016; Guimarães et al., 2018). The results were balanced. Indeed, among the four studies that compared exergaming to aerobic training, two observed a superiority of exergaming to improve cognitive functions, while the three other studies did not. Notably, the two studies that did not observe any superiority of exergaming were MDT studies, which was not expected since MDT included aerobic exercises, but not MCT, and led to enhanced cardio-vascular capacities (Chuang et al., 2015). This is all the more surprising given that in the PCT and MCT studies that have shown superiority of exergaming, the intensity of aerobic effort was low (e.g., Anderson-Hanley et al., 2012). These results are consistent with those observed in their meta-analysis by Soares et al. (2021), who compared the effects of exergames versus conventional physical training on cognitive skills, in both older adults with and without cognitive impairments. They found no differences for attention, processing speed, and executive functions. Only a statistically significant difference in MMSE and MoCA were found among the older people without cognitive impairment, suggesting improvement in global cognitive functioning in favor of virtual reality-based exercises. This latter conclusion was based on the result of only three studies, which were also included in our present work (Bacha et al., 2018; Htut et al., 2018; Moreira et al., 2021). Among these three studies, a qualitative analysis showed that only one (Htut et al., 2018) reported a reliable result for MoCa, while in the two others, no superiority was observed. Even, in these two studies, a passive control group was missing, so that the benefits observed on global cognition, for both exergames and conventional training might rather reflect a test/re-test effect.

• *Exergaming versus cognitive training*

Two studies (Barcelos et al., 2015; Gschwind et al., 2015) showed that the effects of exergaming on cognitive functions strongly depend on the cognitive demands of the gamified environments. However, the two studies (one on MCT and one on MDT) that compared exergaming to cognitive training reported inconsistent results (Eggenberger et al., 2015; Htut et al., 2018). Indeed, Eggenberger et al. (2015) observed superior benefit of exergaming on memory over cognitive (memory) training, while Htut et al. (2018) did not find any superiority after board and card games (i.e., Chinese checkers, Jenga, and Match pair). Although the ‘non-inferiority’ of exergames in comparison to cognitively demanding conditions has been considered elsewhere a positive outcome in itself (Stanmore et al., 2017), these results do not conform to the predictions of some recent models (e.g., the Adaptive Capacity Model; Raichlen and Alexander, 2017) and could cast doubt on the ability of exergames to capitalize on the complementarity of cognitive and physical/motor stimulations Actually, in the absence of a larger number of studies, it remains impossible to draw reliable conclusion.

• *Exergaming versus conventional combined physical training*

Six studies compared MDT exergaming to conventional physical-motor training that is, training programs associating aerobic effort, muscular resistance training and (more or less) complex motor skills (Kayama et al., 2014; Bacha et al., 2018; Htut et al., 2018; Peng et al., 2020; Gouveia et al., 2021; Moreira et al., 2021). In four studies, no superiority of exergaming on cognitive domains was observed, while it was the inverse in the two others for global cognition (Htut et al., 2018) and executive functions (Kayama et al., 2014). Notably, Kayama et al. (2014) used a sequential training procedure to combine conventional training and exergaming within the same session, while Gouveia et al. (2021), using a sequential procedure in two separated sessions, did not observe any superiority of the addition of exergaming to conventional training.

Surprisingly, no study directly compared MDT delivered with exergames neither to conventional MDT nor to cognitive-motor training (dual-task training). However, in their recent review, Gallou-Guyot et al. (2020) carried out an indirect comparison and showed that that training with exergames did not lead to superior benefits than cognitive-motor training via dual-task situations.

• *Comparative efficacy of the different exergame training modes*

Until now, no study compared the benefits of the different exergaming training modes (i.e., PCT, MCT, and MDT, respectively).

• *Effects of exergaming training modes on neurobiological mechanisms and brain functioning*

Three studies (one on MDT and two on MCT) investigated brain activity (Chuang et al., 2015; Eggenberger et al., 2016; Schättin et al., 2016), and one (on PCT) measured blood concentration of BDNF (Anderson-Hanley et al., 2012). Independent of the used exergame, the analysis of brain activity confirmed that exergaming was as effective as conventional aerobic training (but not more) to enhance inhibitory control (Chuang et al., 2015). Also, it showed that both exergame and conventional training reduced brain activity in the prefrontal cortex (PFC), which correlated with improved executive functions and a release of cognitive resources to focus attention on other processes while walking (Eggenberger et al., 2016; Schättin et al., 2016). According to these results the challenge associated with exergames might facilitate activities of daily living, at least when they don’t require dual tasking (for a converging conclusion, Eggenberger et al., 2015; Soares et al., 2021). More generally, possible underlying mechanism at work during exergaming could be that the high cognitive demand of virtual reality may have stimulated more neural pathways, inducing different neurophysiological adaptations than physical training alone (Eggenberger et al., 2016). This hypothesis is consistent with a study that compared virtual reality-based exercises with high versus low cognitive demand, in which challenging exercises were more beneficial for executive functions than less challenging ones (Barcelos et al., 2015; Gschwind et al., 2015).

**FIGURE 3:**
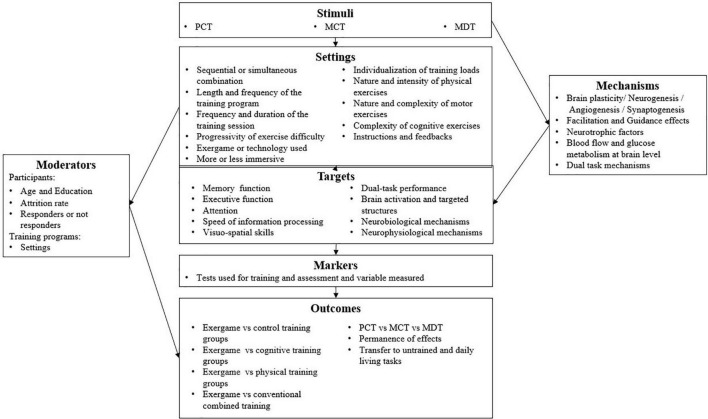
The multi-dimensional model of combined training filled with a brief summary of the findings of the present review.

With respect to the release of neurotrophic factors, it could be that cognitive stimulation had an added value to physical exercise (Monteiro-Junior et al., 2016, 2021), as it has been suggested for conventional combined training interventions (Fissler et al., 2013; Bamidis et al., 2014; Torre and Temprado, 2022). This hypothesis is consistent with the results reported by Anderson-Hanley et al. (2012), according to whom two complementary explanations can be proposed: (i) the physical effort generated by exergames stimulates the peripheral release of neurotrophic factors (mainly BDNF, VEGF, IGF-1, FGF2, and GDNF), which finally cross the blood–brain barrier and enhances neuro plasticity mechanisms and, (ii) gamified environments stimulate brain processes and regions that are activated according to cognitive demands, increasing the release of centrally occurring neurotrophic factors (Monteiro-Junior et al., 2016, 2021). Nevertheless, due to the small number of studies, the mechanisms at work during exergaming are still not fully elucidated and these hypotheses should be confirmed by future works. The conclusions of our analysis are summarized in the Figure 3.

## Strength and Limitations of the Present Work

A strength of the present work is that it analyzed, for the first time to our knowledge, the literature on exergames thanks to a categorization of underlying training modes and a structured framework, instead of according to a “product first” approach. Nevertheless, some limitations include the fact that our analysis focused on brain and cognitive outcomes to the detriment of behavioral, motor and physical outcomes. We adopted this strategy since a number of exergame studies previously addressed separately these aspects. However, since in combined training, cognitive benefits are predicted to be closely linked to physical and motor demands, in future works, it would be interesting to analyze more precisely the relationships between the cognitive and physical/motor benefits observed in the different studies. Unfortunately, in most studies, it was impossible to carry out this analysis, due to the lack of related information. Another limitation lies in the lack of deeper analysis of, on the one hand, the movements really performed by participants (e.g., Skjæret-Maroni et al., 2016) and, on the other hand, of the cognitive contents of the different exergames (e.g., Fronza et al., 2019). This would allow better understanding some moderators of the observed results. However, once again, such an analysis was difficult since this information was scarcely, if any, provided in the different studies. In addition, a deeper division of exergames, based on their level of immersion should be necessary so to distinguish and characterize the effects of more or less immersive and demanding exergames (Huang, 2020; Temprado, 2021).

## Summary and Future Directions

The present work allows concluding that, whatever the type of training (i.e., PCT, MCT, or MDT), exergaming may improve cognitive processes in healthy older adults, which is consistent with the majority of previous reviews (e.g., Stojan and Voelcker-Rehage, 2019; Wollesen et al., 2020). These results suggested that moving (rather than being inactive) brings benefits, which is not surprising according to the widely demonstrated effects of physical activity to prevent age-related declines of functional capacities in healthy older adults.

Less clear was whether there were advantages of exergaming compared to conventional physical, motor or cognitive training. We expected each training mode and their corresponding exergames to yield distinct effects on cognition based on their individual physical, motor and cognitive demand. Indeed, commercial products, like the Nintendo Wii or Xbox Kinect, provide an extensive set of different games, which correspond to varying combinations of physical, motor and cognitive demands. Conversely, virtual ergometers, on the one hand, and dance video game platforms, or step mats, on the other hand, provided other forms of physical/motor activities and cognitive demand. Actually, this hypothesis was not verified. Indeed, in total, among the 11 studies which addressed this question, 8 did not observe any superiority of exergaming over the different forms of conventional training. This result, which was observed independent of the kind of training mode or the used exergames, somewhat contradicts previous reviews (e.g., Stanmore et al., 2017) and the optimism that sometimes prevails about their potential to become the new preventive, anti-aging, medicine (e.g., Monteiro-Junior et al., 2021). On the other hand, it consistent with the conclusion of other recent reviews on exergames (Zhang and Kaufman, 2016; Stojan and Voelcker-Rehage, 2019; Gallou-Guyot et al., 2020; Sala et al., 2021; Soares et al., 2021; Temprado, 2021) and also, those on conventional combined training grounded on a categorization and a structured framework similar to the present work (Torre and Temprado, 2022).

Although PCT, MCT and MDT training modes delivered via different exergames cannot be compared directly, we didn’t find clear differences between the global effectiveness of training modes to improve brain and cognition, neither relative to passive control groups, nor relative to conventional physical, motor or cognitive training. These results are consistent with most earlier reviews on exergames (e.g., Howes et al., 2017; Stanmore et al., 2017; Stojan and Voelcker-Rehage, 2019; Gallou-Guyot et al., 2020; Soares et al., 2021) and, even, on conventional combined training (Torre and Temprado, 2022). In summary, though the different training modes and the corresponding exergames presumably varied in their physical, motor and cognitive demands, this was not reflected in different benefits on cognitive and brain outcomes. Notably, however, as most studies did not systematically report and control physical and cognitive demands, one can only speculate about the levels of the demands of the different training modes and the associated exergames.

Thus, finally, given the limited number of studies, their heterogeneity and the weaknesses of their design quality concerning frequency, intensity and quality of training, more studies are warranted to make more definitive conclusions regarding the ability of exergames to improve cognitive and brain outcomes in older patients. However, this recommendation was found in almost all the reviews published during the last 10 years, thereby suggesting that the new studies filled a bottomless barrel and led to add to the heterogeneity without making significative contributions to better understanding whether and how exergames may bring a real added value relative to conventional training. This situation is highly detrimental since if there is no advantage to using them, it will be necessary for healthcare professionals to rethink the feasibility of such training programs for older patients and/or for the players of the video game market to conceive new products, hopefully more effective to improve brain and cognition. To remedy this situation, two different but complementary avenues are possible. On the one hand, if the existing exergames have the potential to produce beneficial effects, but the studies failed to show it since they poorly conducted (what the present work suggested, in large part), then the scientific community should agree on a consensual protocol that could be carried out in future studies. On the basis of our present analysis and of the Gold Standards proposed in our previous review on conventional combined training interventions (Torre and Temprado, 2022), in the following, we propose some recommendations in this respect (Table 8).

**TABLE 8:** Proposed Gold Standards (GS) identified on the basis of the different constructs of our framework to be considered in future studies to build effective exergaming programs (PCT, MCT, and MDT).

Stimuli	Physical cognitive training	Motor cognitive training	Multidomain training
	•“Training first approach” instead of “product first” (N) • Including a comparison of exergaming + 4 groups (passive control, physical, motor and conventional combined training PCT, MCT or MDT) (HR) • Including comparison between different PCT, MCT or MDT training programs delivered via exergames (R) • Designing training programs of sufficient intensity/complexity to produce effects on cognition and physical/motor abilities (HR) • Assessing systematically the differences between the different training programs (N)		
**Setting**	•Simultaneous combination of cognitive and physical/motor exercises, instead of sequential (HR) • Frequency (2–3 sessions/week) (HR) • Total number of sessions (>12) (HR) • Supervised training by experienced and specialized coaches (N) • Personalization of game choice, and not “off-shelf” proposition (HR) • Individualizing exercise difficulty and complexity (N) • Check that exercises are performed correctly (strategies, priorities…) • Increasing progressively difficulty and complexity (N) • Providing frequent individualized feedbacks (N) • Controlling the level of effort (HR, Borg Rating of Perceived Exertion Scale) (N) • Analyzing the cognitive contents of games (R) • Measuring acceptation, motivation and enjoyment. Comparing with conventional training (HR) • Intention-to-treat + separated analyzes of full completers/partial completers, responders/not responders (R) • Measuring specific outcomes (scores obtained in the games) (R)		
	• Duration of session (45/60 min/session) (HR) • Intensity of aerobic effort (60–80% of Vo2max) (N)	• Duration of session (45/60 min/session) (HR) • Complexity of motor skills: including a large number of degrees of freedom (joints, limbs), requiring control of speed-accuracy trade-off, taxing balance control, perturbing perception (proprioception, vision) (N)	• Duration of session (45/60 min/session) (HR) • Intensity of aerobic effort (60–80% of Vo2max) (N) • Complexity of motor skills: including a large number of degrees of freedom (joints, limbs), requiring control of speed-accuracy trade-off, taxing balance control, perturbing perception (proprioception, vision) (N)
**Target**	•Targeting at least EF (HR), attention (HR), information processing speed (R) and memory (R) using classic laboratory tests • Targeting dual-task performance (R) • Targeting physical capacities (muscular force, muscular resistance, endurance capacities) (N) • Targeting motor capacities (balance, coordination, mobility, agility, psychomotor reaction time) (N)		
**Markers**	•Using different tests for each cognitive function (R) • Using complementary laboratory and field tests to assess physical and motor capacities (R) • Measuring specific outcomes to the games (scores) (R) • Testing permanence of effects and transfer (R)		
**Moderators**	•Age (O) • Gender (O) • Education (R) • Baseline performance level (HR) • Level of immersive environment (HR) • Type of game chosen (HR) • Motivation (O) • Compliance (intention to treat) (R) • Distinction high/low adherers (HR) • Distinction responders/not responders (HR)		
**Outcomes (expected)**	•Significant effects of separated training programs (physical, motor and cognitive) on cognitive performance • Significant effects of separated physical and motor training on physical and motor outcomes • Significant effects of conventional combined training on cognitive, physical and motor outcomes • Larger effect of exergaming compared to corresponding conventional training • Larger benefits of MDT than PCT and MCT. • Permanence and transfer of training		

*Gold standards are recommended to everything possible at best. Accordingly, ideal design features proposed below as Gold Standards were ranked either as: Necessary (N), Highly Recommended (HR), Recommended (R) or Optional (O). N = conditions necessary to ensure the effectiveness of the training. HR = conditions strongly recommended to ensure the quality of the study. R = conditions recommended to increase the interest of the study. O = optional conditions to increase the quality and interest of the study. However, more “realistic” recommendations, with respect to feasibility (i.e., a kind of Minimum Viable Product, MVP) could also be helpful. (Inspired from Torre and Temprado, 2022).*

On the other hand, although gameplay mechanics were scarcely discussed/analyzed adequately in the reviewed studies to be fully understood, we suspected that either the lab-customized or the commercial exergames (e.g., Nintendo Wii, Microsoft Xbox Kinect…) were not appropriately designed for older adults, too complex/difficult, unattractive, and/or not demanding enough in the physical, motor and/or cognitive domains so that game design should be tailored more toward these populations to improve clinical effectiveness of future exergames (for a converging conclusion, see Stojan and Voelcker-Rehage, 2019).

Then recommendations should be made to designed new exergames, hopefully more effective than the existing ones. In particular, they should allow stimulating more heavily the neuromuscular system (counteracting sarcopenia), sensorimotor control and complex coordination, executive functions, multi-tasking, spatial navigation, visuospatial skills and attention exploiting, for instance the theoretical contexts of Evolutionary Neuroscience or Ecological Dynamics (for an extensive development, Temprado, 2021). One can predict that, in the near future, this question will arouse more and more interest in the scientific community, due to the dynamism of companies in the connected fitness market, which develop products very different from those of the players in the video game industry of the early 2000s (e.g., McCaskey et al., 2018; Martin-Niedecken et al., 2019; Martin-Niedecken and Schättin, 2020; Da Silva Júnior et al., 2021; Muńoz et al., 2022).

## Author Contributions

MT and J-JT selected the reviews, meta-analyzes and the studies under consideration, elaborated the structured framework together, analyzed the studies according to the model, and finalized the submitted version. MT wrote the first draft of the manuscript. J-JT contributed to critically revising the initial version. Both authors have agreed with the final approval of the version to be published and agreed to be accountable for all aspects of the work.

## Conflict of Interest

The authors declare that the research was conducted in the absence of any commercial or financial relationships that could be construed as a potential conflict of interest.

## Publisher’s Note

All claims expressed in this article are solely those of the authors and do not necessarily represent those of their affiliated organizations, or those of the publisher, the editors and the reviewers. Any product that may be evaluated in this article, or claim that may be made by its manufacturer, is not guaranteed or endorsed by the publisher.
